# Supplementary material for: Mitigation of PFOA-Induced Developmental Toxicity in Danio rerio by Bacillus subtilis var. natto: Focus on Growth and Ossification
Source: Int J Mol Sci. 2025 Apr 30;26(9):4261. doi: 10.3390/ijms26094261 (PMC12071895; doi:10.3390/ijms26094261)
Supplement: Supplementary file 1 [file ijms-26-04261-s001.zip › ijms-3555671-supplementary.pdf]

Supplementary Materials

Mitigation of PFOA-Induced Developmental Toxicity in *Danio rerio* by *Bacillus subtilis* var. *natto*: Focus on Growth and Ossification

Christian Giommi [1,2,†], Marta Lombó [1,2,3,†], Francesca Francioni [4], Fiorenza Sella [1,2], Hamid R. Habibi [5], Francesca Maradonna [1,2,*] and Oliana Carnevali [1,2,*]

[1] Department of Life and Environmental Sciences, Polytechnic University of Marche, 60131 Ancona, Italy; c.giommi@staff.univpm.it (C.G.); mloma@unileon.es (M.L.); f.sella@pm.univpm.it (F.S.)
[2] INBB—Biostructures and Biosystems National Institute, 00136 Roma, Italy
[3] Department of Molecular Biology, Faculty of Biology and Environmental Sciences, University of León, 24071 León, Spain
[4] Department of Agricultural, Food and Environmental Sciences, Marche Polytechnic University, 60131 Ancona, Italy; f.francioni@pm.univpm.it
[5] Department of Biological Sciences, University of Calgary, Calgary, AB T2N 1N4, Canada; habibi@ucalgary.ca
* Correspondence: f.maradonna@staff.univpm.it (F.M.); o.carnevali@staff.univpm.it (O.C.)
† These authors contributed equally to this work.

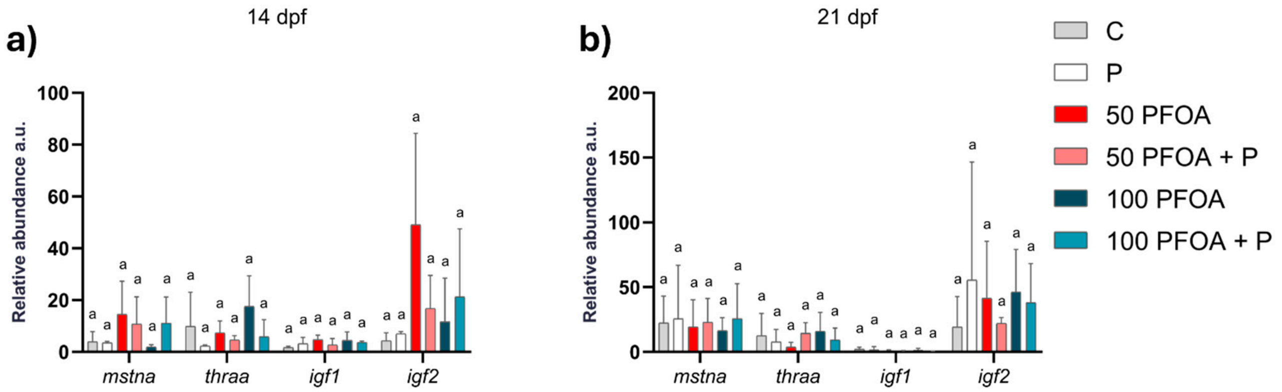

Supplementary Figure S1. Zebrafish growth assessment. Histograms summarizing the *mstna*, *thraa*, *igf1* and *igf2* transcript level in the different experimental groups at **a)** 14 and **b)** 21 dpf reported as relative abundance in arbitrary units (a.u). Data are reported as mean ± SD. Different letters indicate statistically significant changes ($p < 0.05$) among the experimental groups ($n = 5$).

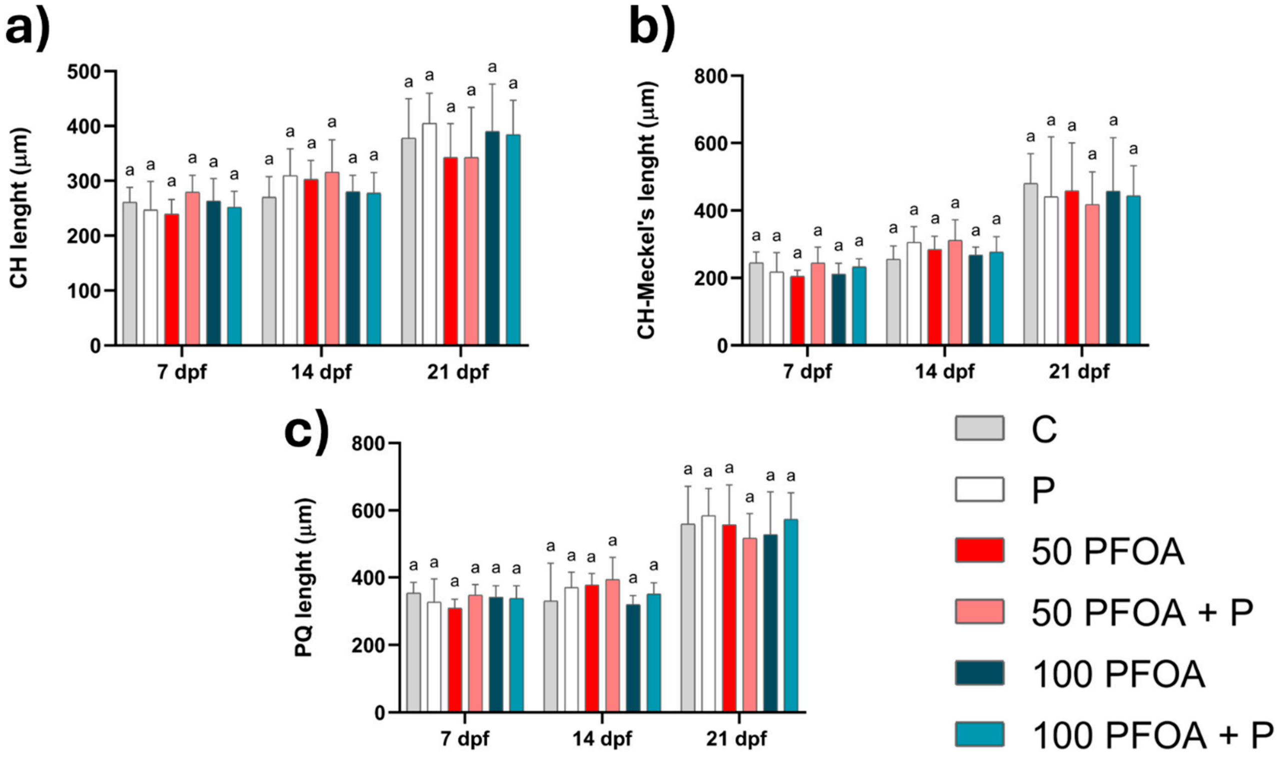

Supplementary Figure S2. Zebrafish craniocephalic morphometric analysis. a) Ceratohyal length, **b)** Ceratohyal-Meckel's length and **c)** Palatoquadrate length in the different experimental groups at 7, 14 and 21 dpf. Data are reported as mean ± SD. Different letters indicate statistically significant changes ($p < 0.05$) among the experimental groups (n = 15) at each timing point.

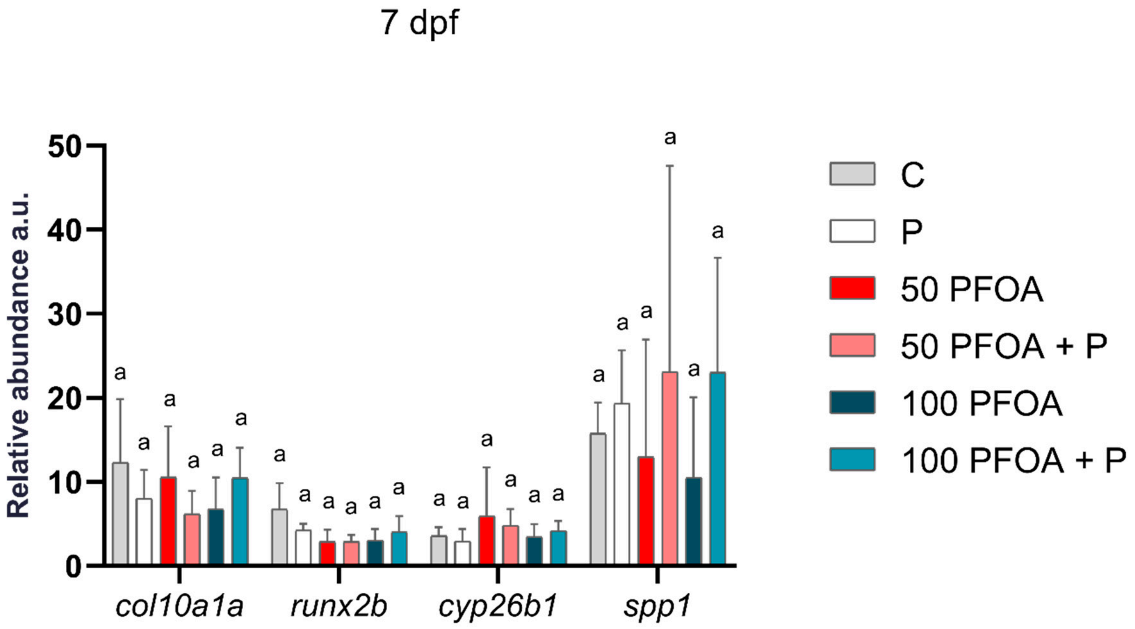

Supplementary Figure S3. Analysis of bone-related genes in zebrafish. Histogram summarizing the *col10a1a*, *runx2b*, *cyp26b1* and *spp1* transcript level in the different experimental groups at 7 dpf reported as relative abundance in arbitrary units (a.u). Data are reported as mean ± SD. Different letters indicate statistically significant changes ($p < 0.05$) among the experimental groups ($n = 5$).

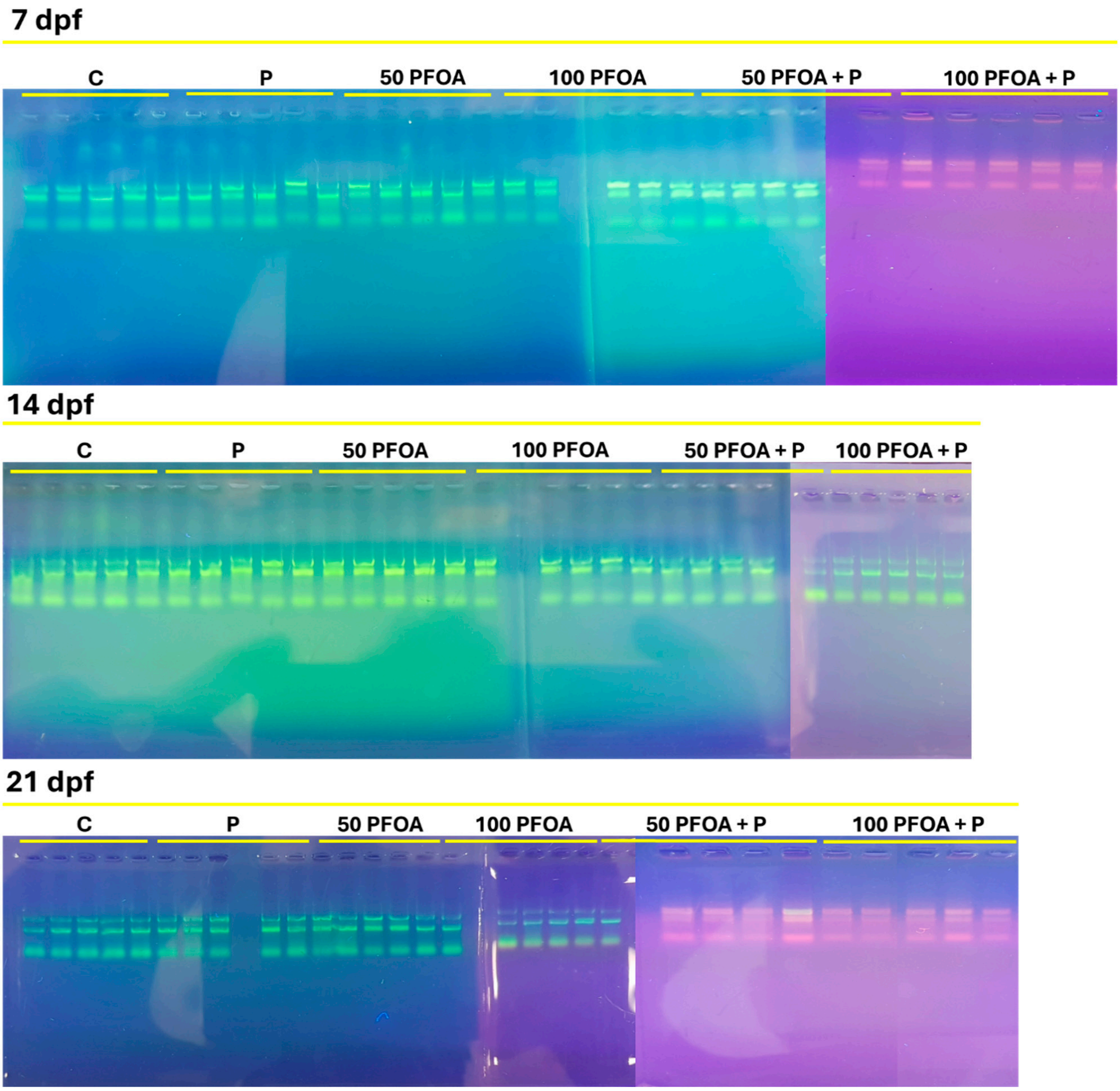

Supplementary Figure S4. 1% Agarose gel run of RNA samples of larvae at 7-, 14- and 21 dpf from each experimental group (5 pools of 25 larvea each per group). RNA were stained by using Fast Red stain or Xpert Green stain.
